# Sustainability of an antimicrobial stewardship program in a community hospital ICU at 3 months post implementation

**DOI:** 10.1186/cc9636

**Published:** 2011-03-11

**Authors:** K Walker, J Sauve, J Powis, V Leung, S Gill

**Affiliations:** 1 Toronto East General Hospital, Toronto, Canada

## Introduction

Our goal was to develop an antimicrobial stewardship program (ASP) and integrate it within a medical/surgical ICU clinical practice. During a 3-month pilot ASP, one pharmacist (Ph) provided clinical service and one antimicrobial (AM) stewardship pharmacist (ASPh) participated in the ICU ASP. Two ASP Phs worked routinely as designated ICU Phms. Post ASP implementation, the ICU Ph added AM stewardship to their role.

## Methods

From 1 April to 30 June 2010, a pilot ASP was implemented in a 490-bed urban community hospital ICU on weekdays. The pilot ASP goals were to optimize/reduce AM usage, improve clinical outcomes and reduce nosocomial *C. difficile *infection rates [1]. The ASPh collected information on ICU patients receiving an AM on a standardized data collection tool. Identified patients were reviewed with the infectious disease (ID) physician, then the ASPh and ID physician met with the ICU care team to discuss ways to optimize AM use. After the pilot ASP, this process was reduced to 3 weekdays and conducted by the ICU Ph, eliminating the ASP Ph involvement. The same metrics used in the pilot program were collected for a 3-month follow-up period [2].

## Results

The pilot ASP resulted in a 47.7% reduction in AM cost from $58,544 (1 April to 30 Jun 2009) to $30,627 (1 April to 30 June 2010). The AM cost in the 3-month post-ASP period (1 July to 30 September 2010) was $22,010. No new cases of nosocomial *C. difficile *infections were identified during the pilot period. Based on an average of 1.4 cases/1,000 patient-days, two cases were expected during the pilot duration. The post-pilot period observed 0.42 cases/1,000 patient-days. The pilot ASP showed a 38.9% reduction of broad-spectrum antipseudomonal AM usage as compared with the same time period of the previous year and a 28.5% reduction in the 3-month post-ASP period. No changes were noted in the Multiple Organ Dysfunction Score or mortality in the pilot and post-pilot groups as compared with the same time period of the previous year.

## Conclusions

The ICU Ph developed the skills required through participation in the pilot ASP program and integrated it within their daily ICU practice. The post-ASP period showed sustained reductions in AM use, costs and nosocomial *C. difficile *rates.
